# More Agility to Semantic Similarities Algorithm Implementations

**DOI:** 10.3390/ijerph17010267

**Published:** 2019-12-30

**Authors:** Kostandinos Tsaramirsis, Georgios Tsaramirsis, Fazal Qudus Khan, Awais Ahmad, Alaa Omar Khadidos, Adil Khadidos

**Affiliations:** 1 Infosuccess3D, 55 Navarxou Kountourgiotou Road, Aigaleo, 122 42 Athens, Greece; 2 Department of Information Technology, Faculty of Computing And IT, King Abdulaziz University, Jeddah 21589, Saudi Arabia; fqkhan@kau.edu.sa (F.Q.K.); akhadidos@kau.edu.sa (A.K.); 3 Dipartimento di informatica, universita’ degli Studi di Milano, 20122 Milan, Italy; Awais.Ahmad@unimi.it; 4 Department of Information Systems, Faculty of Computing And IT, King Abdulaziz University, Jeddah 21589, Saudi Arabia; aokhadidos@kau.edu.sa

**Keywords:** Gene Ontology similarity algorithms, GO semantic terms similarity, gene/gene product semantic similarity, digital health

## Abstract

Algorithms for measuring semantic similarity between Gene Ontology (GO) terms has become a popular area of research in bioinformatics as it can help to detect functional associations between genes and potential impact to the health and well-being of humans, animals, and plants. While the focus of the research is on the design and improvement of GO semantic similarity algorithms, there is still a need for implementation of such algorithms before they can be used to solve actual biological problems. This can be challenging given that the potential users usually come from a biology background and they are not programmers. A number of implementations exist for some well-established algorithms but these implementations are not generic enough to support any algorithm other than the ones they are designed for. The aim of this paper is to shift the focus away from implementation, allowing researchers to focus on algorithm’s design and execution rather than implementation. This is achieved by an implementation approach capable of understanding and executing user defined GO semantic similarity algorithms. Questions and answers were used for the definition of the user defined algorithm. Additionally, this approach understands any direct acyclic digraph in an Open Biomedical Ontologies (OBO)-like format and its annotations. On the other hand, software developers of similar applications can also benefit by using this as a template for their applications.

## 1. Introduction

Many methods have been developed for the execution of Gene Ontology Semantic Similarities algorithms, but none of them allows the user to define and execute user-defined algorithms. Instead they offer to the user the ability to choose from a static list of pre-implemented algorithms. These implementations have a fixed number of algorithms that the user can apply to a predefined dataset. The potential of the current implementation approaches is limited since they only allow execution of a pre-defined number of GO semantic similarity algorithms. 

In this paper, we are going to address the problems of defining and executing new semantic similarity algorithms, as well as loading different types of annotation files, rather than the standard Gene Ontology Annotation (GOA) [1]. This will allow researchers to shift the focus to the execution of the algorithms rather than coding the algorithm itself. To deal with the aforementioned issues, we propose an implementation approach (see Supplementary Materials) to allow users to define GO semantic similarity algorithms, load any annotation file, and execute it. The main novel contributions of this paper are: 1) Dynamic algorithm definitions: mounting and execution, and 2) directed acyclic digraphs loading through Dynamic Parsing.

The former one is the most important contribution in this paper. It enables new, user-defined algorithms to be generated dynamically. Additionally, as part of this research we have developed a tool (see Supplementary Files) that implements the proposed approaches. In this tool, we follow a question/answer approach where the user will “teach” the program how to use the new algorithm by answering a number of questions. The tool will then store and execute this algorithm. All algorithms and results are stored in text files, for future references.

The latter one will allow users to load any kind of acyclic digraph in similar Open Biomedical Ontologies (OBO) file format and annotated it though another file. If the annotation file is not in the same format as the GOA annotation file, the user must “teach” the software how to read and use the annotation file by following a question and answer approach.

In the next section we review a number of implementations of GO semantic similarity algorithms. In the third section, we describe the proposed approach in detail. In the fourth section, we evaluate the approach by demonstrating how a popular algorithm such as Resnik [2] can be defined dynamically. In the fifth section, we briefly discuss about gene/gene product similarities. In the sixth section, we show how any directed acyclic digraph can be loaded through dynamic parsing. Finally, in the seventh section, we summarize the contribution of our work to GO semantic similarity and propose future perspectives.

## 2. Related Work 

To the best of our knowledge there is not any current approach to provide to users the ability to define different GO semantic similarity algorithms. One of the most recent approaches was developed by Schlicker and Albrecht [3], which developed the FunSimMat tool. They have developed an implementation that was able to find Semantic similarities and give the output in a matrix. The users were only able to find similarity only from a standard dataset and apply only well-established algorithms like Resnik [2], Lin [4], and Jiang Conrath [5]. The tool is not able to load new OBO files and allow the user to apply a pre-defined algorithm to the data set. 

Another tool that was reviewed was the ProteInOn, developed by Faria et al. [6]. ProteInOn is able to perform algorithms such as Lin [4], Resnik [2], and Jiang Conrath [5], which are used to compare different methods for finding GO semantic similarities. ProteInOn cannot support adding a new algorithm or loading new OBO files. 

Apart from the two tools that we reviewed there are many others that deal with gene/gene product and GO term similarities. Some of them are web-based, some of them are stand-alone applications and some of them are packages than run under other programs such as Matlab. According to Pesquita et al. [7] most of them provide services and information, such as protein interactions (ProteInOn), GO browsing (DynGO), graph visualization (GOvis, DynGO), and clustering (GOToolBox, G-SESAME, csbl.go). None of them, however, support dynamic algorithm definition. 

Almasoud et al. [8] discusses about the current biological SSMs (semantic similarity measures) that they cannot handle big data. In order to manage big data a three-step method was proposed, i.e. data clustering, split gene ontology, and semantic similarity calculation to lever big data. Yu [9] proposed a method for automatic indexing of Medical Subject Headings (MeSH) in Medline/PubMed. Gene set enrichment analysis is also provided using MeSH annotation to extract the biological meanings from the gene list, expression profile and genomic regions. Yang et al. [10] presents a solution to the missing values in the microarray data profiles, by connecting microRNA (miRNA) with gene ontology through miRNA-targeted genes. An overview of biomedical text mining tools and bioinformatics applications was provided by Lamurias and Couto [11], and it was proposed that algorithms should be developed to turn text into a structured representation. Scientific literature poses a challenge to methods of text mining as the vocabulary used is formal and highly specialized. Asgari et al. [12], proposed a distributed vector representations of segments of biological sequences called ‘biovectors’, using a neural network of skip-grams. They suggest an intrinsic assessment of biovectors by evaluating the consistency of the underlying biophysical and biochemical properties (e.g. average weight, hydrophobicity, and charge). Liu et al. [13] suggest that biases in the available GO annotations result in also biased estimates of gene functional similarities. However, it remains unclear what the impact of incompleteness may be, even in the absence of bias; both "complete" and "incomplete" data sets of GO annotations from the GO database were proposed for the gene functional similarities. Zhao and Wang [14] developed an algorithm, GOGO, that calculates semantic similarities using GO DAG topology. The algorithm can calculate similarities between multiple pairs of GO terms and genes. It can classify genes based on functional similarities. However, it does support user defined algorithms. 

Both ProteInOn and FunSimMat were very useful for the researchers that were working on standard datasets, but on the other hand, they were found unable to perform semantic similarity using user-provided annotation files. Also, they could not be used by those researchers who were not using Gene Ontology or the ones who were using another method. Nevertheless, these tools are very likely to become obsolete, since they are not adapted to support the new methods that will be developed in the future. In this paper, we are going to address the aforementioned problems by proposing a mechanism that allows users to define and execute their own algorithms. Our method would allow researchers to focus on the development of improved algorithms rather than the implementation of a given algorithm. Additionally, our approach would be suitable for application on any OBO file.

## 3. User Defined GO Semantic Similarity Algorithm Definition and Execution

**Figure 1:**
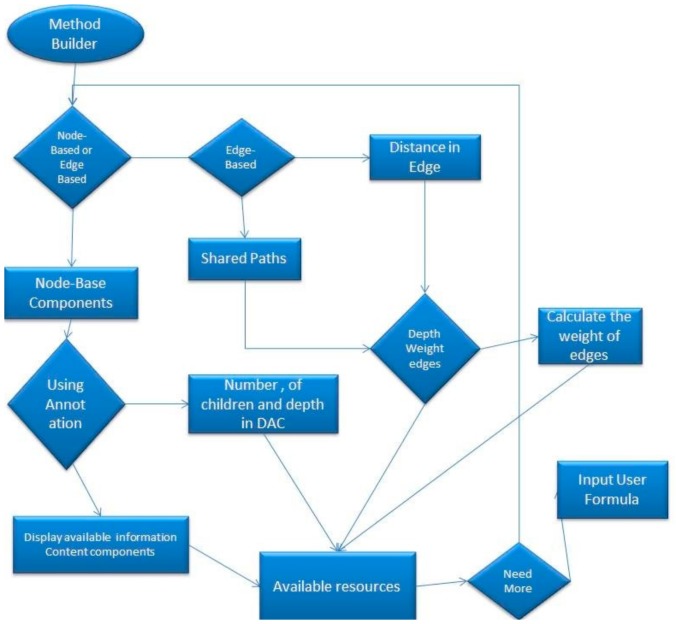
Selection of algorithm components.

According to Pesquita et al. [7], GO semantic similarity algorithms are broadly classified into ‘node-based’ and ‘edge-based’. Node-based approaches rely on comparing the properties of the terms involved, which can be related to the terms themselves, their ancestors, or their descendants [7] and edge-based approaches are based mainly on counting the number of edges in the graph path between two terms [15]. Node-base approaches are further divided into those that use annotation (information content) [16] and to those that do not. The proposed approach supports both node-based and edge-based algorithms. More specifically, the tool can support Information Content approaches, which are applicable only to Direct Acyclic graphs. Furthermore, a component of those approaches is the depth of a term in the DAG and with edge-based algorithms such as Distance algorithm. Figure 1 represents the process for the selection of algorithm components by a user in order to create a user-defined algorithm.

First, the user selects the components, either Node-based or Edge-based, that are going to be used. It is worth mentioning that the user is able to come back to this stage and choose both node-based and edge-based components. The user has to select whether components will use the Information Content algorithms, like Resnik’s algorithm, annotation, together with Direct Acyclic Digraphs (DAG) or just the DAG itself. In case the user chooses to use annotation, then the Information Content components will receive the following components; *term1, term2*, and Common Ancestor. All of the above terms will be explained in detail in the section followed, with an example of how a user-defined algorithm can be built. If the user chooses not to use annotation, then the following components will be received: The depth of the nodes, the depth of the Common Ancestor and the number of child nodes of each node.

When the user chooses the edge-based components, it will be requested to select the Shared Paths (the number of edges from the common ancestor until the root) or the distance between the two nodes, which are being compared. Next, the user has to select the shortest or the average distance. Additionally, the user can retrieve the total or the maximum distance, in case a new algorithm needs to be defined. After that, the user has to decide between depth of the weighed edges and edges with weighed value one. 

The selected components are stored in a list. The tool then requests a formula which combines the chosen components. This formula consists of mathematical operations such as log, square root, subtract, divide, add, raise to a power of the number of components provided.

## 4. Detailed Example of an Algorithm Loading

**Figure 2:**
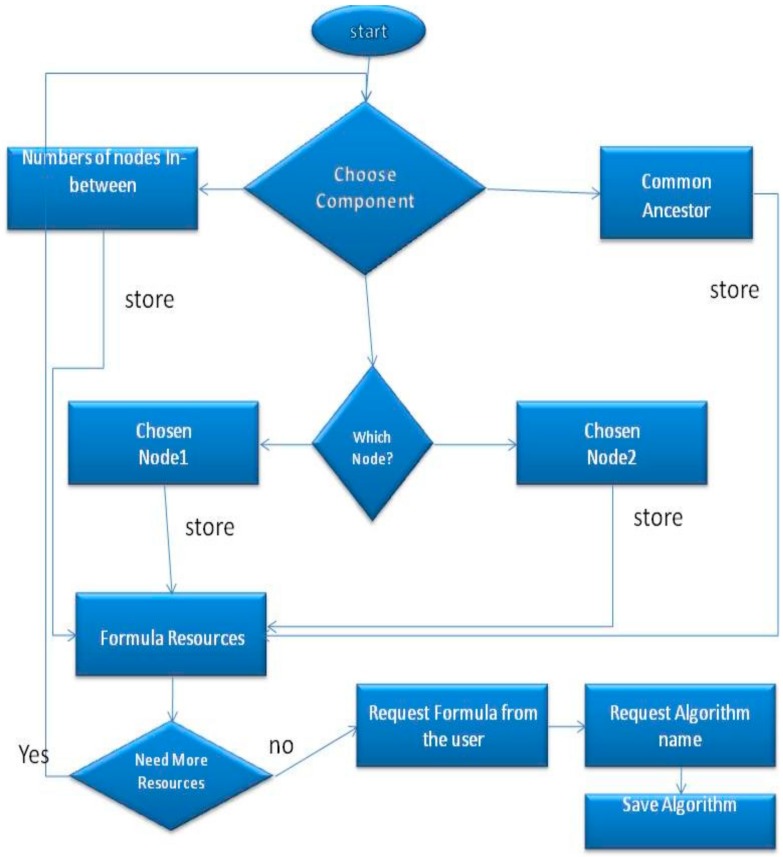
Information content components.

In this section we will provide a detailed description of the algorithm. At the end of this section we will also offer a “Computer–User” question–answer example. This part is enabling the users to define their own GO semantic similarity algorithms and execute them for finding similarities in the directed acyclic digraphs. The user will be asked to answer to a series of questions, providing in this way all necessary information to the tool to design the algorithm. More specifically, the user will be able to apply any mathematical operation on the common ancestor or on the nodes that are compared. The overall process is illustrated in Figure 2.

First, the user has to specify which components will be used (Figure 2). The available components are the following; P (the probability of occurrence of each term, which can be found in the annotation file) of the actual nodes that are compared, P of the common ancestor, all the parent nodes of the nodes that are compared, all nodes’ child nodes and children of the common ancestor and their number P. Next, the user will create a formula using the components that were chosen above from an available resource for this formula (e.g., in Resnik similarity the only component that the user should use for the *compare* operation is the Common Ancestor, in order to specify the Resnik formula in which case the Log of number P is the similarity). Thus, the user will be able to apply a mathematical calculation (like Log) in the Number P of this component. In addition, our implementation can count the number of the nodes that are in between, so the user can apply a mathematical formula on them as well.

The Semantic Similarities Measures are used to carry out various tasks. To the best of our knowledge, the most popular Semantic Similarities Measures include Resnik [2], Lin [4], Jiang et al. [5]. All these methods are Information Content Methods [2]. In this paper we chose Resnik for its simplicity and we demonstrate how users can define their own algorithms. 

In Resnik, the probability number is used to find similarities between terms. The probability can be extracted from an annotation file, because each gene product of the annotation file refers to a term within the DAG. In Resnik, the similarities are detected by applying -Log of the probability number of the common ancestor that can be found from the Annotations file:

*Resnik(term*1*, term*2*)=−Log(PCom)*

where PCom is the number P of the common Ancestor. The common Ancestor is the term that contains both terms (term1, term2) as its children. The proposed approach, allow users to define their own algorithms. In the following example we illustrate how the Resnik algorithm can be defined in our system using a question and answer approach.

Comp: 1.Which node-based component you want to use?
Comp: (Press the corresponding number 1.Common Comp:Ancestor, 2.Node1, 3.Node2,4.Distance)
User: 1
Comp: 2. Do you need any other component?
Comp: You already have: Common Ancestor
User: No
Comp: Please Write your formula, for the probability number write P dot and the component for Comp:example:
Comp: (for Node one: P.node1, a formula should look like that log(P.Node1))
User: log(P.CommonAncestor)
Comp: Please give us the name of the formula
User: Resnik
Comp: Please confirm the following:
Comp: Name Resnik
Comp: Formula: -log(P.CommonAncestor)
User: Yes
Comp: Your Algorithm has been saved and is ready to be used

The above example demonstrates how the user can define a GO semantic similarity algorithm, such as Resnik. This tool allows users to define any algorithm in a similar way, eliminating the need for programming knowledge. In this way, the user focuses more on the solution of real-world problems, rather than computer programming. In the next section, we will discuss how genes can be translated to terms.

## 5. Gene Products and Their Translation to Terms

**Figure 3:**
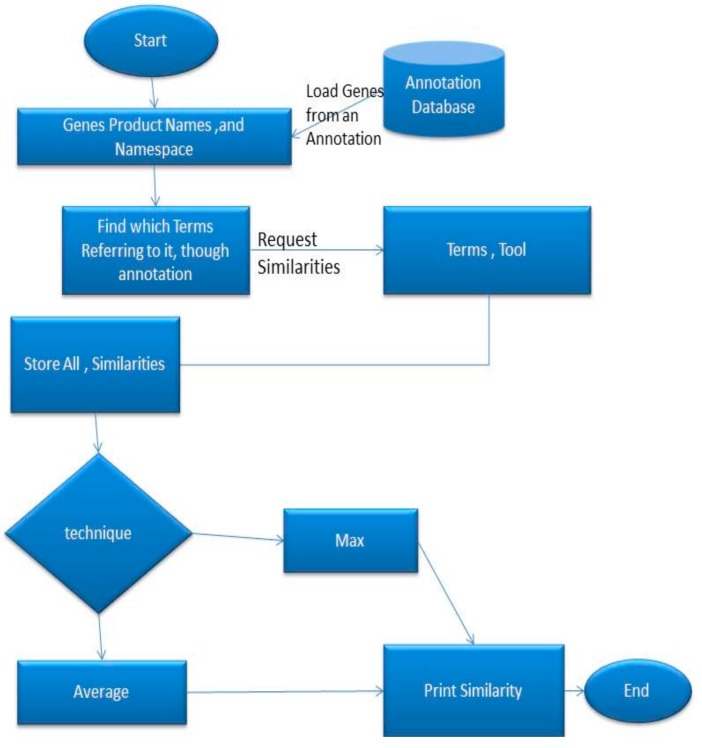
Translation of gene products to gene terms.

One way to find similarities in two gene/gene products is by detecting their corresponding term similarities. The terms are separated in name-spaces. The proposed tool is fed with the name-space information in order to guide the tool to estimate a correct similarity. The procedure is presented in the Figure 3:

As shown in Figure 3, the user feeds the algorithm with the gene’s product names and a name space. The algorithm then translates the gene’s product to terms. The terms that are not in the user define name space are ignored. Next, the algorithm sends these terms to the term tool, which seeks the similarities with the procedure that we defined previously. The term tool then returns an array with all the similarities. The gene tool then requests from the user if the max or the average technique will be used. The Max method calculates the maximum similarity between two gene products, and the average estimates the average from the similarity array. It is worth noting that this gene/gene product similarity method is a pair-wise approach, as it has been described by Pesquita1 et al. [7].The proposal presented in this paper does not support group-wise approaches.

## 6. Import Custom Direct Acyclic Graphs

**Figure 4:**
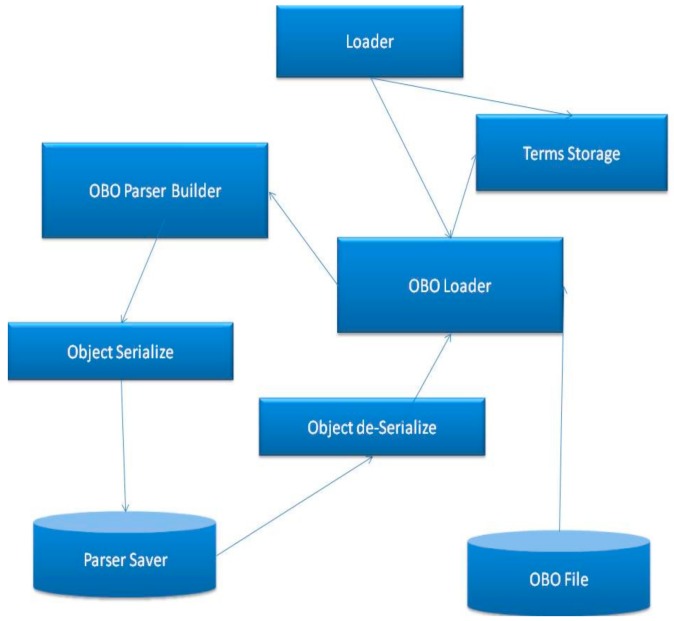
Open Biomedical Ontologies (OBO) file parsing.

The focus of this paper is the implementation of user-defined GO semantic similarity algorithm, however in this paragraph we are going to describe a generic parser, which can accept any type of OBO files rather than simply GO OBO files. This is because the bioinformatics community often uses OBO files, and thus it is more useful to implement such a generic parser. OBO files are structured as Directed Acyclic Graphs (DAG), where the nodes represent terms and the edges indicate different types of relationships between them [17]. Taking this in account, we made an effort to make our approach more generic by applying the user-defined algorithm on preloaded DAGs and their corresponding annotation files. This additional implementation allows users to use standard or user-defined algorithms on any OBO file associated with any annotation file. The procedure of loading OBO and annotation files is illustrated in Figure 4.

The tool first loads the OBO file and creates a DAG. Then, it loads the annotation file and annotates each term in the DAG. In order for the parser to identify a non-GO OBO file, the user should determine the format of the file by providing at least an ID, a name and Parents list for each node in the acyclic digraph. 

An OBO file contains gene ontology terms. Every term has a Name a GO id, definition and a namespace it may also have a relationship. Following we can see the signs that indicates what exist in the lines. Note: This list contains only the elements that we use for parsing. The remaining sings and keywords were ignored as they were out of scope.

- [Terms] means that a new term, is starting
- id: means that this line contains the GO id
- name: this line contains the name of the GO
- def: this line contains the definition
- namespace: this contain the namespace of that the Go belongs
- is_a:this line contains a “is_a “the relationship
- relationship: part_of: this line contains a “part_of“ the relationship

The following is an example of a term, as it appears in OBO files.

[Term]
id: GO:0000001
name: mitochondrion inheritance
namespace: biological_process
def: “The distribution of mitochondria, including the mitochondrial genome, into daughter cells after mitosis or meiosis, mediated by interactions between mitochondria and the cytoskeleton.” [GOC: mcc, PMID:10873824, PMID:11389764]
synonym: “mitochondrial inheritance” EXACT []
is_a: GO: 0048308 ! organelle inheritance
is_a: GO: 0048311 ! mitochondrion distribution

**Figure 5:**
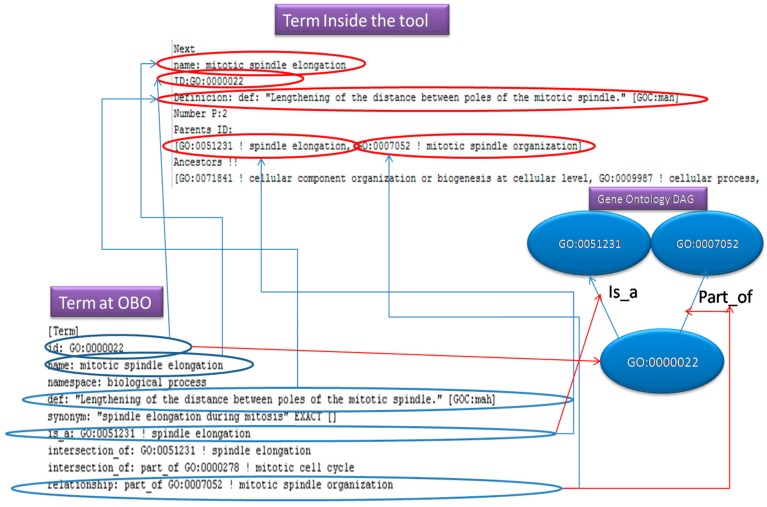
OBO entries.

The diagram in Figure 5, presents how an OBO entry is been store in an OBO file. Additionally, it illustrates, how the fields of this entry, correspond to the term (object) fields inside our program and what they represent into a Gene Ontology DAG. The “id: “ is the node, the “is_a” and “part_of” lines indicate the parent nodes.

The OBO parser will generate an object with the following fields; id: String, name: String, Parents_Relationship: Array List of Strings and comment_definition: String as mandatory fields. Additionally, the system receives some optional fields; Description: String and Namespace: String. After the user feeds the above information to the new object, it will be serialized and stored. Then the parser object will be loaded dynamically on demand from the serialized location (file, database, memory, and so on), when it is required to parse a file. 

The OBO parser is parsing the OBO files by reading every line of the file and if the line starts with “user-defined id” then it stores the value of the ID in a temporary term object. Then, it is searching the text for the values of the mandatory and optional fields defined above, until it finds the next ID or until it finds the end of the file. After storing the values, the temporary term is stored to an array list that will store all the terms of the directed acyclic digraph. All terms contain a field that hosts the *p* (probability) value. After parsing, the tool will create an ancestor list for each term. The ancestor list will be populated by their ancestors’ IDs recursively. After finding all ancestor IDs for all terms, the tool will load the annotations file. Once the OBO file is loaded, the tool will load the selected annotation file. The latter should be divided by lines or by columns and contain IDs, which are going to be used when annotating the nodes. There are two different procedures that the user has to follow, depending on whether it is parsing lines or column. If it is parsing lines, then the user has to specify the gene ID and the term ID that are contained in the line (for example, ‘GO: xxx’ for terms or ‘UniProtKB: xxx’ for gene products in the Gene Ontology annotation), as well as the alphanumerical numbers following these IDs (for example, 7 for GO terms). Then, the user may specify which lines should be ignored by defining some characteristics (like the evidence code), and whether these characteristics precede or follow the term ID (on the line). Moreover, the user must define the comment signs in the annotation file, so that these lines will be automatically ignored.

Parsing the columns works in a slightly different way. The user has to define the column containing the elements that will be used to annotate a node in the acyclic digraph (for example, the GO: id) and the column that contains the gene product ID. Then the user is requested again to provide the comments sign and the column that contains elements that if found the whole line should be ignored. Once the user has specified these elements, the parser will be ready to start. After the procedure is finished, the user will have his/her own parser. Afterwards, data can be parsed by applying the default methods for comparison or a customized method on the data for both annotation loaders. If the parser finds an ID of the DAG, the tool finds where the direct acyclic graph is stored in the array list and adds 1 to the Number P for this node and their ancestors. In order to calculate the ancestors, we start with the first term that we load in the OBO loading step; we backtrack from the parents of this term until the root recursively. We add each term that we found, until the root in a list that will keep the ancestors for this term. After that a term will know its entire ancestor. At this stage we will have a complete tree that it is ready to perform any edge-based Gene Semantic Similarity method. In the next step we utilize the annotations. 

**Figure 6:**
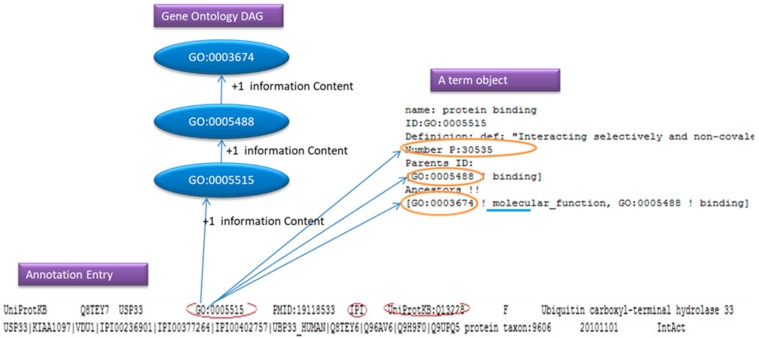
An annotation example.

For simplicity reasons we can think of an annotation file as a number of entries that consist of few lines containing a GO: id, evidence code and a Gene Product. As we will see in Figure 6, the annotation parser adds one to the information content of the GO: id and its ancestor. If the user has request to ignore the evidence code, and the parsers find the evidence code in the line, the line will be ignored.

In Figure 6, the GO: 0005515 is the GO that we found in the annotation entry, the IPI is the evidence code and the UniProtKB: Q13228 is the Gene Product.

To annotate the Ontology, the user has to feed the program, with an annotation file (GOA). The Annotation File contains annotation reference, this reference will help as annotate the Gene Ontology object and will also help as to populate a Gene Product Storage that will hold Genes names.

Annotation References contain a Gene Name, a single reference of a go, evidence code, and other information that we will not need for this report such as taxon in the database. 

**Figure 7:**
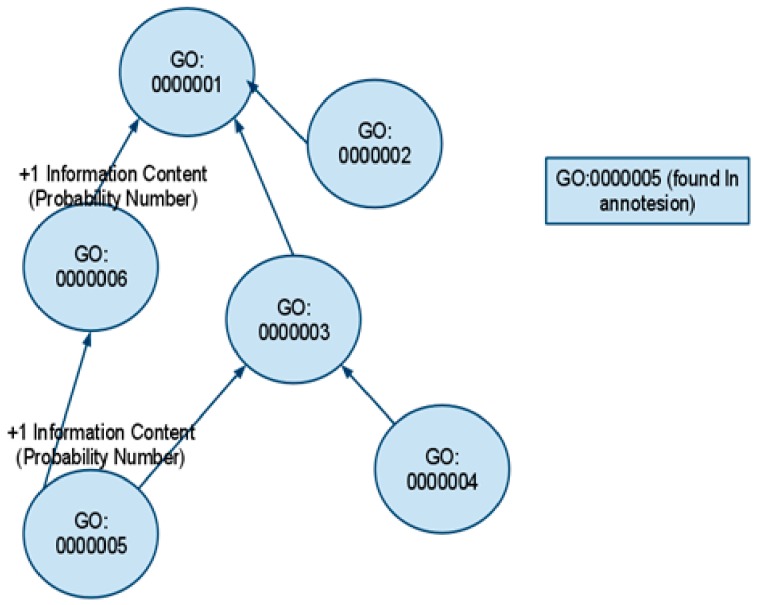
Gene Ontology Tree.

The Annotation parser will read line by line and every time that will find a GO: id in a line it will annotate the GO term at the Gene Ontology, adding one to the Information Content number (probability number) of the term that has been found on the annotation file. All the ancestors of this term are also loaded. Example results of this process are presented in Figure 7 as a gene ontology tree.

In this example, we found number GO: 000005 on the annotation file and we add one, to GO: 0000005, GO: 0000003, GO: 0000006, and GO: 0000001 probability number. The data is ready for both Edge- Based and node based Gene Ontology methods. 

**Figure 8:**
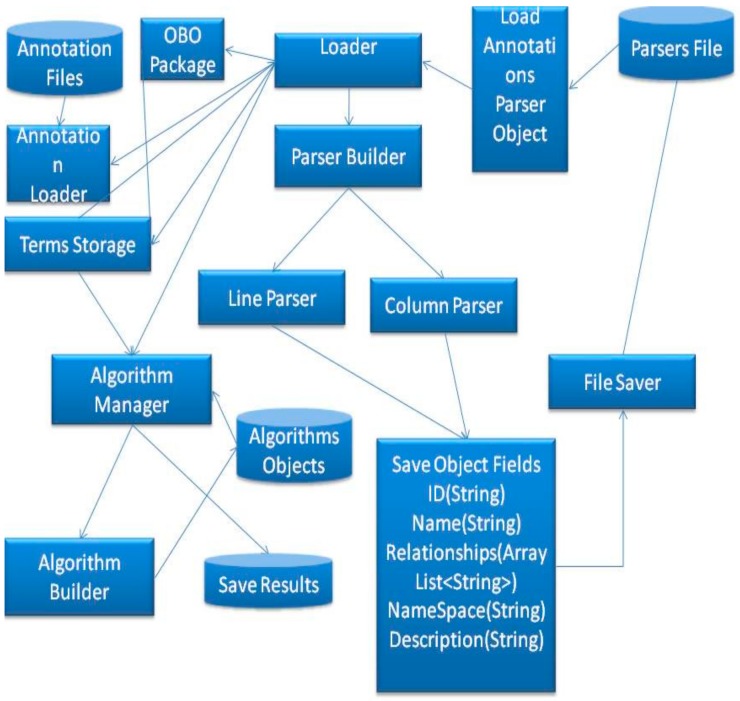
Implementation flow.

To summarize the section, Figure 8 illustrates the connections and shows the structure of the proposed mechanism. 

As shown in Figure 8, the proposed method involves loading the parser object from files, a file that contains a parser can be created by a user, including the following information; numbers of columns for the annotation elements, number of columns for gene IDs, number of columns for ignoring lines and the comments sign for this annotation. The tool will save this data into a parser object and send this object to the loader. The loader will send the data to the annotation Loader which will start parsing the file line by line and annotate the DAG, which is stored in terms Storage. After that, the terms storage will be ready to execute semantic similarity search algorithms.

The work was evaluated using a software engineering approach called black-box testing [18]. In this approach, the testers provide the system with inputs for which the expected outputs are known. If the actual outputs match the expected outputs then the system pass the test. During the evaluation phase, we developed 10 test cases that cover all the basic functionality of the system. The proposed implementation passed all the test cases. 

## 7. Conclusions

In this paper, we introduce a proposal for making semantic similarity algorithm implementations more flexible by supporting user-defined algorithms. We aim to help the bioinformatics community to focus on developing and improving semantic similarities algorithms and we offer a more elegant and user-friendly way for doing it. Also, this tool is intended to allow each user to add an implementation that is not included and share it with the rest of the community. Another feature that we propose is parsing any OBO file through a user-defined dynamic parser. This enables the community to make available any kind of direct acyclic digraph and an annotation file to enable comparison of some other type of data apart from Gene Ontology. In conclusion, we believe that our proposed computational method would allow users to focus on the development, improvement and execution of their algorithms rather than coding. 

Currently, “link density" is not supported for node-based algorithms. Algorithms of this category will be supported in the next proposal. Additionally, we plan to set up an online repository that will enable users to upload their user-defined algorithms. This can serve as a depository of knowledge for the research community. This database can be linked with other databases such as the Gene Ontology Annotation (GOA) database, which is the central source for gene/protein-GO term annotations [1]. Additionally, we will provide an API that will allow other tools to be connected with the database. At the current stage, the users define their algorithms by answering a number of questions where they enter the keywords, which the tool will look for in an OBO file. In the future we plan to replace this function by an interface that will be able to add components visually and apply algorithms on them, similar to visual programming languages. This will address usability issues and make the program more approachable to a bigger variety of users.

## Supplementary Materials

The implementation of the proposed approach is available online at https://github.com/fazalqudus/GO-Similarity-Tool.
